# Elucidation of the evolutionary expansion of phosphorylation signaling networks using comparative phosphomotif analysis

**DOI:** 10.1186/1471-2164-15-546

**Published:** 2014-07-01

**Authors:** Hisayoshi Yoshizaki, Shujiro Okuda

**Affiliations:** Department of Pathology I, Kanazawa Medical University, 1-1 Daigaku, Uchinada, Ishikawa, 920-0293 Japan; Department of Biomedical Sciences, College of Life Sciences, Ritsumeikan University, 1-1-1 Noji-higashi, Kusatsu, Shiga, 525-0058 Japan; Graduate School of Medical and Dental Sciences, Niigata University, 1-757 Asahimachi-dori, Chuo-ku, Niigata, 951-8510 Japan

## Abstract

**Background:**

Protein phosphorylation is catalyzed by kinases and is involved in the regulation of a wide range of processes. The phosphosites in protein sequence motifs determine the types of kinases involved. The development of phosphoproteomics has allowed the identification of huge numbers of phosphosites, some of which are not involved in physiological functions.

**Results:**

We developed a method for extracting phosphosites with important roles in cellular functions and determined 178 phosphomotifs based on the analysis of 34,366 phosphosites. We compared the conservation of serine/threonine/tyrosine residues observed in humans and seven other species. Consequently, we identified 16 phosphomotifs, where the level of conservation increased among species. The highly conserved phosphomotifs in humans and the worm were kinase regulatory sites. The motifs present in the fly were novel phosphomotifs, including zinc finger motifs involved in the regulation of gene expression. Subsequently, we found that this zinc finger motif contributed to subcellular protein localization. The motifs identified in fish allowed us to detect the expansion of phosphorylation signals related to alternative splicing. We also showed that the motifs present in specific species functioned in an additional network that interacted directly with the core signaling network conserved from yeast to humans.

**Conclusions:**

Our method may facilitate the efficient extraction of novel phosphomotifs with physiological functions, thereby contributing greatly to the analysis of complex phosphorylation signaling cascades. Our study suggests that the phosphorylation networks acquired during evolution have added signaling network modules to the core signaling networks.

**Electronic supplementary material:**

The online version of this article (doi:10.1186/1471-2164-15-546) contains supplementary material, which is available to authorized users.

## Background

Protein phosphorylation is a ubiquitous post-translational modification, which controls several cellular processes in cell signaling networks. Cellular protein phosphorylation is catalyzed by protein kinases, which are one of the largest gene families in eukaryotes. The disruption of cellular protein phosphorylation events causes several diseases, including cancer and diabetes, and kinase inhibitory molecules may have potential uses in targeted molecular therapy to combat certain diseases. The human genome encodes 518 protein kinases, which are divided into nine groups based on their sequence similarities, including one tyrosine kinase family and eight serine/threonine kinase families [1]. Several protein kinases have local substrate specificities that are determined by the amino acid sequence surrounding the phosphosite as a kinase motif. The techniques used for protein phosphorylation analysis have advanced rapidly since the development of high-throughput phosphoproteomic analysis, which has yielded extremely large phosphopeptide datasets [2–4]. Techniques and algorithms have also been developed for bioinformatics analysis that combine large-scale phosphoproteomics datasets with other information from a wide variety of life science databases to predict signal transduction networks, thereby leading to a better understanding of cellular functions [5, 6]. However, it is difficult to extract useful information related to cellular functions based on phosphosites alone because it is necessary to understand both the kinase protein and the functional change in its substrate protein caused by phosphorylation. Thus, a large number of phosphosites have been reported, but only a small number of kinase–substrate relationships have been identified. Therefore, several studies have attempted to develop methods to predict these relationships [7–11].

Previous analyses of intracellular signaling networks used methods that subtract the subnetworks identified in various conditions, such as cell cycles and ligand stimulation, or in different species [12]. Similarity methods such as evolutionary comparative analyses of phosphosite sequences have been used for phosphorylation signaling network analysis [13–16]. The position of a phosphosite in a protein is affected by both its primary structure and the corresponding kinase, which may be changed by evolutionary processes [1, 17]. In general, the amino acids in a protein have evolved at a constant rate from yeasts to humans. In the present study, we assume the evolutionary order of model organisms on the basis of the evolutionary distances from the viewpoint of humans, as described previously [18, 19]. Phosphorylated serine/threonine amino acids have an evolutionary rate similar to that of other amino acids and they exhibit rather low conservation [20, 21]. However, proteins with constrained specific amino acid sequences, such as R-X-X-S/T, rather than a phosphorylated serine alone, are likely to be evolutionarily conserved [22]. These conserved motifs probably play important roles in cellular functions [23–25]. Thus, comparative evolutionary analyses based on sequence evolution are powerful tools for extracting phosphorylation signals that are directly linked to cellular functions. Previous comparative evolutionary analyses of phosphorylation have focused on conserved sites for exploring the important signaling networks that are considered to be essential for cellular functions [14, 16, 20] and for the rewiring of signaling networks [13, 21, 24, 25].

However, focusing on highly conserved sites would not allow us to extract the highly evolutionarily conserved signaling networks that are essential for cellular functions because additional signal transduction pathways have been acquired throughout evolution; therefore, low conservation sites are also important in cellular functions. However, it is also well known that several low conservation phosphosites do not have physiological functions. Previous comparative analyses could not distinguish these differences; therefore, we developed a method for investigating evolutionary patterns to determine the peripheral components that have been acquired throughout evolution. We analyzed a large number of phosphosites and simplified the complex phosphorylation signaling networks. The same motifs located in different proteins are likely to be phosphorylated by a kinase that belongs to a closely related protein family and these motifs may be useful for identifying candidate kinases responsible for phosphorylation [26]. Thus, we identified motifs as putative signaling modules added during specific evolutionary processes. We demonstrate that our method is a useful tool for analyzing and understanding intracellular signaling networks, thereby determining the regulatory mechanisms of cellular processes.

## Results

### Extraction of phosphomotifs

Previous studies attempted to construct phosphomotifs for each kinase [27]. However, these studies focused on less than half of the known kinases, and the phosphomotifs are still not available to facilitate a comprehensive analysis of phosphorylation. By contrast, the information related to phosphosites has been updated and an analysis based on bioinformatics was published recently. To investigate the evolution of phosphomotifs, we extracted phosphomotifs from publicly available databases for phosphosites. Phosphomotifs comprise the downstream and upstream regions that together form a phosphosite [26]. We extracted three types of phosphomotifs, i.e., X-X-X-X-X-pS/T/Y, X-X-X-pS/T/Y-X-X-X, and pS/T/Y-X-X-X-X-X, from 97,679 phosphosites in PhosphoSitePlus [11]. We calculated the similarity scores between them by adding the score in the BLOSUM62 substitution matrix at each sequence position, as described previously [20]. The sequence pairs with a similarity score >9 were clustered using the MCL [28] to generate motifs that corresponded to three motif sequence patterns. Thus, we obtained 474 clusters and manually extracted 178 phosphomotifs by identifying the conserved amino acids in each cluster (Additional files 1 and 2). These motifs covered 77% (75550/97679) of the phosphosites used in the clustering analysis. They also included 57%, 51%, and 68% of all the serine, threonine, and tyrosine residues, respectively, obtained from all human proteins.

### Comparative evolutionary analysis of the phosphomotifs

We investigated the evolutionary conservation of the phosphosites in each motif to elucidate the physiological roles of the identified motifs. To analyze the evolutionary conservation, we selected model organisms with rich genome information, i.e., *Saccharomyces cerevisiae* (fission yeast), *Schizosaccharomyces pombe* (budding yeast), *Caenorhabditis elegans* (worm), *Drosophila melanogaster* (fly), *Danio rerio* (fish), *Canis familiaris* (dog), *Mus musculus* (mouse), *Pan troglodytes* (Chimpanzee), and *Homo sapiens* (human). We obtained orthologous gene sets from KEGG OC [29] for the nine species. We constructed multiple sequence alignments of phosphoproteins in each orthologous group and identified species where a known phosphosite was conserved in a motif. We counted the number of proteins with phosphomotifs (Additional files 3-A and 4). In addition, we counted the conservation of the serine/threonine/tyrosine residues observed in all human proteins as potential phosphosites. The number of conserved residues between evolutionarily neighboring species increased to a maximum of only approximately 30% between the fish and dog in the case of serine/threonine residues, and approximately 32% between the worm and fish in the case of tyrosine residues (Additional file 5). Using the conservation rates of known and potential phosphosites, i.e., serine/threonine/tyrosine residues observed in all human proteins, we defined a conservation index to estimate the evolutionary conservation of phosphomotifs. The conservation index was calculated as the sum of the differences between the conservation rate of a known phosphomotif and that of the corresponding known and potential phosphomotifs in all species (Additional file 3-B). This conservation index represented the conservation of a motif relative to the average amino acid conservation.

**Figure 1:**
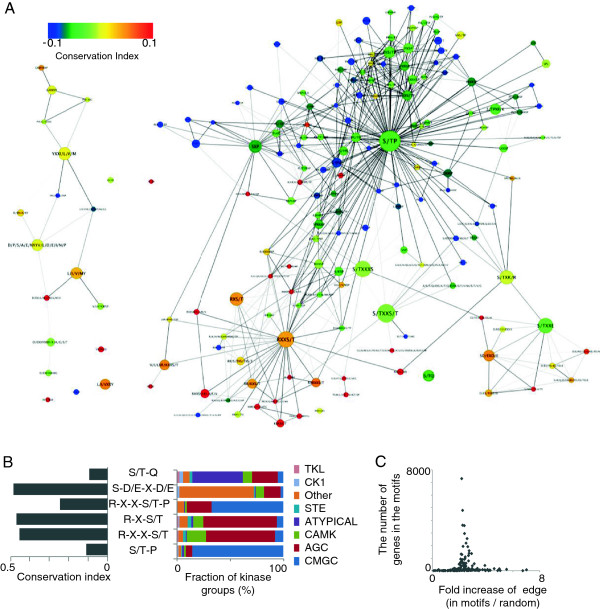
**Comparative evolutionary analysis of the phosphomotifs. (A)** Network diagram showing the sequence motif patterns and their conservation. The motifs were connected if the protein sequences that included the motifs were shared in more than 20% cases. The node colors that represent each motif are based on the conservation indices, ranging from blue to red. The network was created using Cytoscape. **(B)** Relationships between the conservation of motifs and the kinases that phosphorylated proteins with the motifs. The annotations of the kinase group names were determined on the basis of the known kinase–substrate relationships obtained from phosphorylation databases. Well-known motifs observed in several proteins were selected. **(C)** Increases in the interactions related to genes in phosphomotifs. The fold increase in the number of known interactions for genes in a motif was compared with that for random genes.

Our 178 motifs included similar motifs, where only one or several amino acids differed. We investigated the correlation between the motif structures and the evolutionary conservation of our phosphomotifs, and we generated a network of motifs on the basis of their structural similarities (Figure 1A). We observed that the motifs with proline adjacent to serine/threonine (S/T-P) were found often in MAPK phosphomotifs with a low level of conservation, whereas the motifs (R-X-X-S/T, R-X-S/T, etc.) with an arginine residue upstream of the phosphosite were often observed in the phosphomotifs of AGC kinases, and they were highly conserved. It is well known that the phosphomotifs used as substrates by kinases are related to the directional characteristics of kinases [26]. To investigate the correlations between the conservation indices and kinase families of our motifs, we extracted the known kinases from PhosphositePlus; Figure 1B shows the correlations. We observed that 86% of the kinases that phosphorylated the low conservation S/T-P motifs belonged to the CMGC family. However, 69% of the RXS/T motifs and 65% of the RXXS/T motifs were phosphorylated by the AGC kinase family. These results suggest that the phosphorylating enzymes correlated with the motif structures and conservation level. To characterize the phosphomotifs, we investigated the protein–protein interactions with the motifs using known protein interaction information from BioGRID [30] and STRING [31]. The number of edges in an interaction network that contained the phosphomotifs was 2.5 times larger than that in a randomized dataset (Figure 1C), which suggests that proteins with a phosphomotif are likely to be connected with each other.

**Figure 2:**
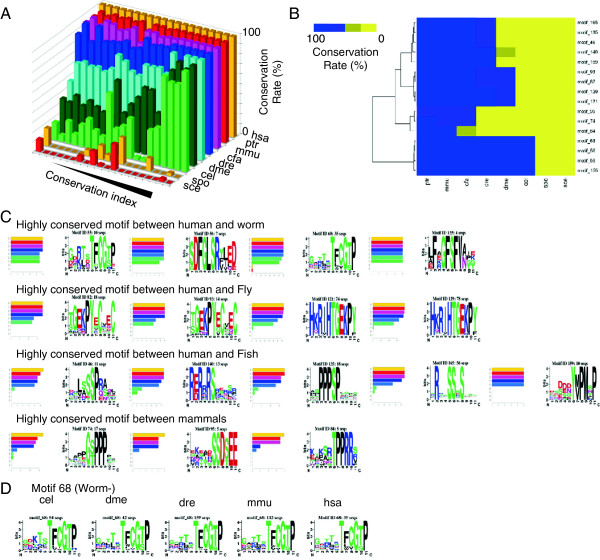
**Extraction of phosphomotifs with sigmoid-type evolutionary patterns. (A)** Conservation rates of phosphomotifs. The top 20 motifs with high conservation index values are listed. **(B)** Clustering of the evolutionary patterns of the phosphorylation motifs. Motifs where the conservation rates changed >50% between two evolutionarily neighboring species were selected. **(C)** List of the motifs that acquired phosphosites from a specific species. Bar plots and sequence logos of the motifs from the worm, fly, fish, and dog are listed. **(D)** Example of sequence conservation in a motif across species. The sequence logos in motif 68 are shown for species ranging from the worm to humans. Abbreviations: hsa (*Homo sapiens*), ptr (*Pan troglodytes*), mmu (*Mus muscullus*), dre (*Danio rerio*), dme (*Drosophila melanogaster*), cel (*Caenorhabditis elegans*), spo (*Schizosaccharomyces pombe*), and sce (*Saccharomyces cerevisae*).

Figure 2A shows the conservation of the phosphomotifs and their conservation indices. We identified two different patterns for the phosphomotifs with high conservation indices. One was highly conserved in all species and its evolutionary conservation curves exhibited a relatively linear pattern. The other was highly conserved in humans and specific species where the evolutionary conservation curve exhibited a sigmoid pattern (see motifs B and C in Additional file 3A). We designated the former pattern as “linear” motifs and the latter as “sigmoid” motifs. It is assumed that the conservation of motifs exposed to evolutionary pressure will be dramatically higher among closely related species, thereby yielding sigmoid conservation patterns.

The conservation of known phosphosites decreased slightly from humans to yeasts in a similar manner to the conservation of serine/threonine residues as if the motifs had not been exposed to evolutionary pressure. However, the motifs subjected to evolutionary pressure had a sigmoid-type pattern with dramatic changes among species (Figure 2B and C). This suggests that the phosphorylation of motifs with sigmoid patterns is essential after speciation and that they originated from functional differences between species and their ancestral species (Additional file 3). We extracted sigmoid-type motifs on the basis of a threshold value using the average rates of transitions and their standard deviations (Figure 2B), which identified motifs 55, 56, and 68 as those that increased after worm speciation; motifs 82, 93, 121, and 129 for the fly; motifs 46, 135, 140, 159, and 160 for the fish; motifs 95 and 74 for the dog; and motif 84 for the mouse (Figure 2C). To visualize the conservation relationship among known phosphosites and their motifs, we generated sequence logos [32]. The sequence logos confirmed that evolutionarily conserved phosphosites also contained highly conserved flanking regions (Figure 2D and Additional file 6). In addition, we investigated the conservation of phosphosites and the proteins containing these sites (Additional file 7), which detected the conservation of phosphosites related or unrelated to protein conservation.

### Protein networks of highly conserved motifs in humans and the worm

**Figure 3:**
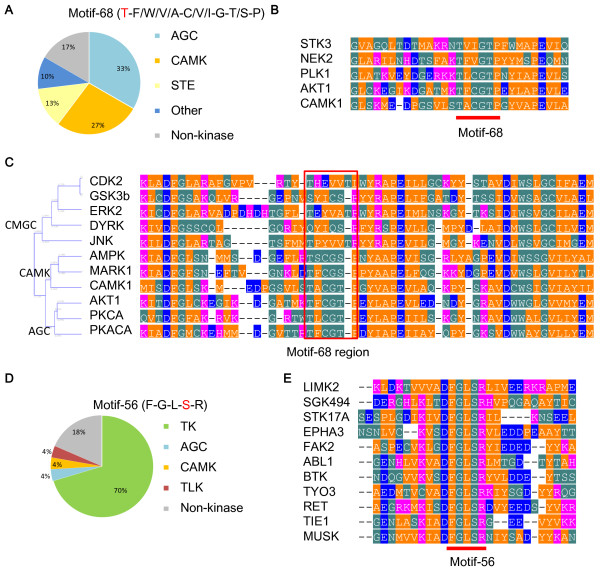
**Highly conserved motifs with known and potential phosphosites present within the protein kinase domain in species ranging from humans to the worm. (A)** Pie chart showing the distribution of kinase families, including motif 68 (motif 55 is included in motif 68) in humans. **(B)** Sequence alignment of the flanking regions of the proteins, including motif 68, with their phosphosites. The red bar indicates motif 68 regions. At least one sequence was selected in each kinase group. **(C)** Sequence alignment of the AGC, CAMK, and CMGC proteins, including motif 68 with AGC and CAMK protein phosphosites. The red box indicates motif 68 regions. The phylogenetic tree was drawn using the UPGMA method. **(D)** Pie chart showing the distribution of protein functions, including motif 56 in humans. **(E)** Sequence alignment of the flanking regions of the proteins, including motif 56 with its phosphosites. The red bar indicates motif 56 regions. At least one sequence was selected in each kinase group.

We observed that motifs 55, 56, 68, and 155 were highly conserved in the fish and humans. Motifs 55 and 68 shared eight sequences with quite similar sequence logos. These phosphomotifs were located within a kinase domain and they exhibited high conservation (Figure 3A and B). In addition, the phosphosites in these motifs were highly conserved in the worm in known phosphomotifs and in the same motifs identified in all proteins (Additional file 8A). Motifs 55 and 68 were observed in 36 serine/threonine kinases, i.e., 56%, 25%, and 11% in the AGC kinase, CAMK, and STE kinase families, respectively (Figure 3A). For motif 56, we observed that 70%, 15%, and 15% were assigned to the tyrosine kinase, S/T kinase, and non-kinase families, respectively. We found that CMGC proteins could be aligned with AGC and CAMK proteins in the sequence regions without motif 68 (Figure 3C). The phosphosites in AGC kinase family proteins with motifs 55 and 68 have been reported to be phosphorylated by PDK1, a serine/threonine kinase [33]. We compared motifs 55 and 68 with the phosphomotifs for PDK1. We confirmed that there was a highly conserved position of five amino acids downstream of the phosphorylated serine/threonine sites (Additional file 8B). In addition, the loss of PDK1 was lethal in species ranging from yeasts to mouse, which demonstrates that PDK1 is essential for the fundamental cellular activities [34–36]. The results suggest that a number of orthologous groups may be substrates for PDK1, which increased after worm speciation. Of these phosphorylated motifs, 30 motifs were conserved in the worm, whereas three, two, and one sequences were conserved in the fly, fish, and fission yeast, respectively. Most examples of motifs 55 and 68 were located in serine/threonine kinase domains, whereas most examples of motif 56 were located in tyrosine kinase domains recognized by tyrosine kinases such as the Eph receptor, PTK, and BTK (Figure 3D and E). Kinases that phosphorylate motif 56 have not been reported.

### C2H2 zinc finger proteins from highly conserved motifs in humans and the fly

**Figure 4:**
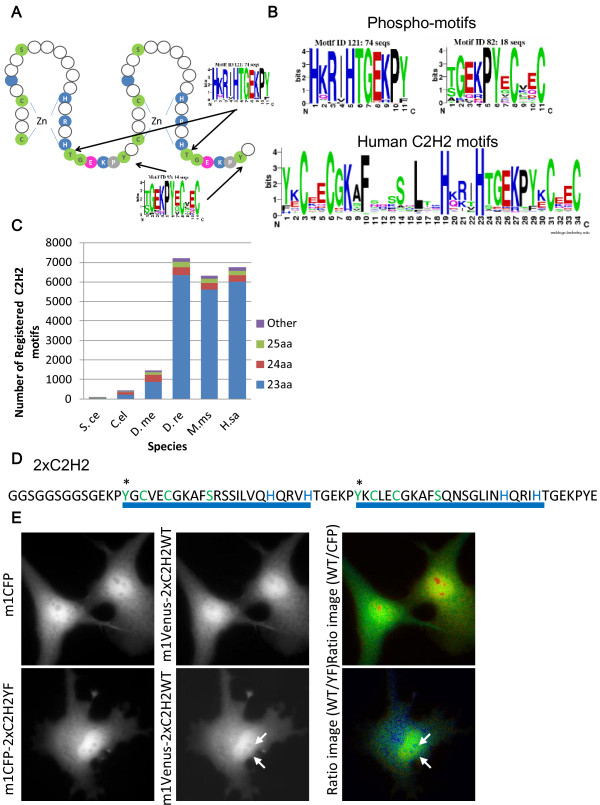
**Highly conserved motifs present in C2H2-type zinc finger motifs that regulate C2H2 localization in humans and the fly. (A)** Structure of the C2H2-type zinc finger motif and the positions of motifs 82, 93, 121, and 129. **(B)** Sequence logos of the C2H2 motifs observed in all human proteins and those conserved in humans and the fly. **(C)** Number of C2H2 motifs observed in the genomes of species ranging from yeasts to humans. The colors in the bar plot indicate the lengths of the C2H2 motifs. **(D)** C2H2 motif sequence synthesized on the basis of the C2H2 motif in human ZNF24 protein. The ZNF24 proteins are known to be phosphorylated at tyrosine and threonine residues. The asterisk indicates a phosphosite. **(E)** Localization of m1Venus-2xC2H2 and m1CFP in Cos7 cells. The image showing m1Venus-2xC2H2 relative to m1CFP was produced to demonstrate their localization (upper images). Localizations of m1Venus-2xC2H2 and the m1CFP-2xC2H2YF mutant in Cos7 cells. The image of m1Venus-2xC2H2 relative to m1CFP-2xC2H2YF was produced to demonstrate their localization (lower images). The 2xC2H2YF mutant with a YF mutation at the tyrosine residue, which is indicated by an asterisk in Figure 4E. The arrows indicate nuclear locations with higher relative differences in CFP and YFP images.

Motifs 82, 93, 121, and 129 were observed to be highly conserved motifs in species ranging from the fly to humans. Motifs 121 and 129 shared most of the proteins related to each motif, although these motifs were different. Motifs 82 and 93 also shared several of these proteins. A network of 194 proteins obtained from these motifs was extended using interaction information obtained from BioGRID and STRING, similar to Figure 2B, but only nine proteins were connected by known interaction information (data not shown). We investigated the domain structures of these motifs using Pfam search and observed that all phosphomotifs were related to a zinc finger motif (Figure 4A). The C2H2 motif is a structure where two cysteines and two histidines chelate zinc and proteins and has multiple tandem zinc finger motifs that can bind to DNA [37]. The C2H2 motifs were observed primarily in transcription factors, and over 6000 C2H2 motifs have been identified in the human genome [38]. We detected 6743 C2H2 motifs in our dataset. It has been reported that zinc finger proteins with C2H2 motifs regulate development and cell differentiation [39]. This motif was conserved in species ranging from the fly to humans, where a serine between cysteines and histidines, a threonine immediately downstream of the second histidine, and a tyrosine two amino acids upstream of the first cysteine in the C2H2 motif were phosphorylated.

To investigate the conservation of phosphosites in human C2H2 motifs, we produced the sequence logos of known C2H2 motifs. We observed that the tyrosines in motifs 82 and 93 and the threonines in motifs 121 and 129 were highly conserved in each motif (Figure 4B).

Four phosphomotifs that increased after fly speciation were related to a C2H2 motif; therefore, we investigated whether the evolutionary conservation of the phosphomotifs and all C2H2 motifs were correlated (Figures 4C and 2C). We observed that the number of C2H2 motifs increased after fish speciation, whereas the conservation of phosphomotifs increased after fly speciation, which suggests two different hypotheses: proteins that possess C2H2 motifs without phosphomotifs have increased selectively since fish speciation, and proteins with a C2H2 motif related to phosphorylation have been duplicated since fish speciation. To test these hypotheses, we investigated the frequencies of all the phosphomotifs. In the human genome, 3749, 1423, and 2507 phosphomotifs were observed in motifs 82, 93, and 129, respectively. The human genome contained a total of 6743 phosphomotifs and 56% of them were related to motif 82, which was considerably higher than the number of C2H2 motifs that increased in the fly. This suggests that proteins in the human genome with C2H2 motifs have been duplicated from the proteins that increased since fly speciation. The increase in C2H2 motifs in fish may reflect an increase in retroelements because the C2H2 motifs in zinc finger proteins have been evolutionarily duplicated by retroelements [40]. Thus, we investigated the sequence differences between all C2H2 and phosphorylated C2H2 motifs. We extracted a sequence that started two amino acids upstream of the first cysteine until the last histidine as the overall C2H2 motif. The C2H2 motif sequences were classified into three groups depending on their lengths, i.e., 23, 24, and 25 amino acids. We observed that 94% of the C2H2 motifs belonged to these three groups, where 90% of these were 23-amino acid C2H2 motifs (Additional file 9A). These three C2H2 motifs differed from the 23-amino acid-type motif in terms of the presence of only one and two amino acid insertions between the histidines in the 24- and 25-amino acid-type C2H2 motifs, respectively, and there were no other amino acid changes (Additional file 9-B). We investigated the differences between all C2H2 motifs and the phosphorylated 23-amino acid C2H2 motifs. The 1st tyrosine was present in 70.3% of all the 23-amino acid C2H2 motifs, and the 24th threonine was present in 68.9% of the C2H2 motifs. Therefore, these two amino acids could be universal in C2H2 motifs.

C2H2 zinc finger motifs are fundamental functional motifs in species ranging from yeasts to humans. We generated sequence logos for all C2H2 motifs coded by organisms from yeasts to humans (Additional file 9-C). In the first sites of motifs 82 and 163, a fraction of the tyrosines had increased throughout evolution, although phenylalanines dominated in yeasts. It has been reported that a phosphorylated threonine in motifs 121 and 129 affects the interactions between the C2H2 motif and DNA sequences, thereby regulating the cell cycle [41, 42]. These previous reports indicate that comparative motif analysis is a powerful tool for extracting biologically important phosphomotifs. We focused on motif 82, for which the regulation of phosphorylation has not been reported. To investigate whether the phosphorylation of motif 82 is related to the functions of C2H2 motifs, we generated oligo sequences that mimicked C2H2 motifs, i.e., 2×C2H2-YF, where we replaced Y with F in motif 82, and 2×C2H2-SN, where we replaced S with N in motif 163, which had the smallest evolutionary effect as a control sequence, based on the ZNF24 sequence containing a phosphorylated C2H2 motif for motifs 82 and 163 (Figure 4D). 2×C2H2 labeled with mCFP was transfected into Cos cells, and 2×C2H2 localized to the nucleus and cytosol. 2×C2H2 was not likely to have localized to the nucleus because of the loss of the SCAN domain and NLS [43]. As a control, we observed localization when fluorescent proteins were co-expressed. m1Venus-2×C2H2 was highly localized to the nucleolus compared with mCFP. We compared the localization to the nucleolus of 2×C2H2 and 2×C2H2-YF (Figure 4E). We observed that the YF mutant was localized to the nucleolus at a higher level compared with the wild type. This suggests that the phosphorylation of motif 82 in the wild type inhibits localization to the nucleolus and that the tyrosine in motif 82 regulates the localization of C2H2 motifs because the YF mutant exhibited strong localization to the nucleolus.

### Regulatory pathways of RNA splicing and insulin signaling based on highly conserved motifs in humans and the fish

**Figure 5:**
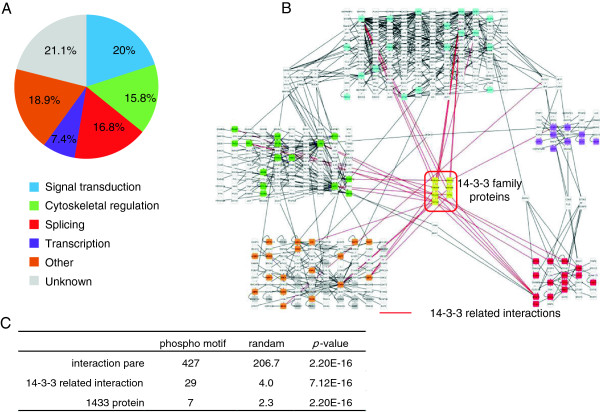
**The highly conserved motifs in humans and the fish have signal modules related to splicing, insulin signaling, and cytoskeletal regulation. (A)** Summary of the functions of the proteins, including fish motifs. The functions were classified using the UniProt annotation information. **(B)** Signaling network of proteins with motifs 46, 135, 140, and 165, which were highly conserved in humans and the fish. The node colors indicate the functions defined in Figure 5A. Yellow nodes indicate 14-3-3 proteins and blank nodes indicate the nodes added by the network expansion method. The network structure is grouped into protein functions, which are clustered based on their interactions with the functional proteins. The network was created using Cytoscape. **(C)** Statistics related to the interaction network of fish phosphomotifs. The numbers of edges and nodes related to 14-3-3 proteins were counted. Random interaction networks were generated 100 times using the same number of proteins with the phosphomotif.

Motifs 46, 135, 140, 159, and 165 were highly conserved in species ranging from humans to the fish. To explore whether the phosphomotifs were correlated with specific physiological protein functions in the motifs described above, we performed functional enrichment analysis based on gene ontology (GO) [44] for proteins with known phosphosites (Additional file 10). Motif 159 increased after fish speciation, and the results suggested that it was correlated with cell migration and insulin signaling proteins. In addition, motif 140 appeared to be present in proteins related to RNA processing and RNA splicing. We observed that 95 proteins included these motifs and their functional categories were classified using Uniprot annotation information (Figure 5A). In addition, motif 140 was observed in several RNA splicing components. Motif 159 was observed in adapter proteins that play a role downstream of the growth factor signals, such as insulin signaling. Approximately half of the proteins with these five motifs were classified into three functional groups: insulin signaling molecules, RNA splicing-related factors, and cytoskeleton regulation factors. Subsequently, we investigated the connectivity of these motifs in molecular interaction networks. The number of proteins with known phosphomotifs was considerably small, and it was difficult to assign correct biological functions using a network that only connected them; therefore, we produced an interaction network related to proteins, including phosphomotifs, using known interaction information from BioGRID and STRING. The interaction networks reconstructed using each motif that increased after fish speciation included signal transduction, RNA splicing, and cytoskeleton-related proteins (Figure 5B). We obtained an interaction network with 427 edges using 95 protein nodes. The 14-3-3 proteins in this network had significantly more interactions compared with a randomly generated network with the same number of nodes (Figure 5C). The network reconstructed using these five motifs had a network structure where 14-3-3 kinases linked three pathways: splicing, cytoskeleton regulation, and insulin signaling. The 14-3-3 family proteins bind to phosphoserine-containing proteins and motifs phosphorylated by AGC and CAMK family members [45, 46]. In humans, seven subfamilies of the 14-3-3 protein family have been reported, and they dramatically increased after fish speciation, whereas only two are known in the fly. The 14-3-3 proteins may be involved in protein phosphorylation signaling, and the evolution of 14-3-3 proteins and phosphomotifs may be correlated. In addition, it has been reported that insulin signaling pathways induce neuronal survival via alternative splicing of the protein kinase CdII isoform [47]. The splicing-related proteins and signal modules of the insulin signaling-related proteins that emerged after fish speciation may have co-evolved.

### Addition of the signaling network of phosphomotifs with sigmoid conservation patterns to the core signaling network led to the acquisition of new cellular functions

The phosphomotifs that appeared after fish speciation were components of the signaling networks related to fundamental functions such as the cytoskeletal regulation, signaling, and splicing. These networks are common signaling networks, but it is considered that these functions have probably been conserved since before fish speciation. These results suggest that the motifs that have increased since fish speciation may be related to the expansion of the signaling networks. Thus, we investigated the conservation of all known phosphosites in our motifs using human proteins from these signaling networks.

**Figure 6:**
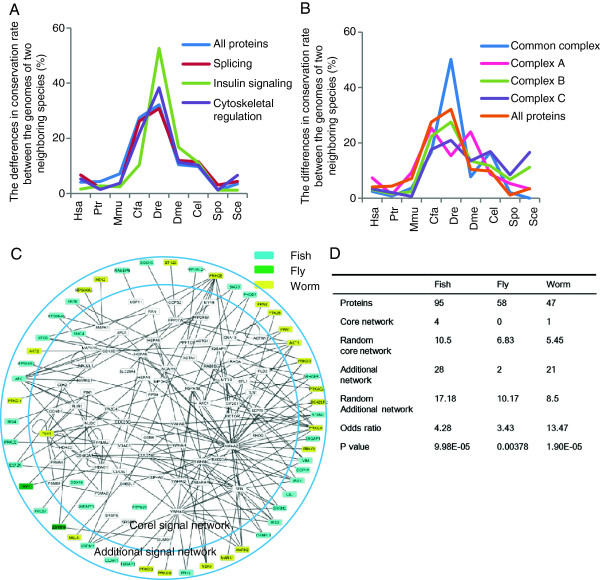
**Evolutionary expansion of the core signaling network and acquisition of new cellular functions. (A)** Differences in the conservation rates of phosphosites between two evolutionarily neighboring species in each category, including all proteins, spliceosomal proteins, cytoskeletal proteins, and insulin signaling-related proteins. **(B)** Differences in complexes A, B, and C, and common components of spliceosomal proteins. **(C)** Core signaling network of the proteins with sigmoid-type phosphomotifs (motifs 55, 56, and 58 for the worm; motifs 82, 93, and 121 for the fly; and motifs 46, 135, 140, 159, and 165 for the fish) and the additional network. Blue, green, and yellow boxes indicate proteins with sigmoid-type motifs in the fish, fly, and worm, respectively. **(D)** Statistics for the core signaling network and additional network. Random interaction networks were generated 100 times using the same number of proteins with the sigmoid-type phosphomotifs in each genome. Odds ratios were calculated for the additional to core signaling network. The odds ratios in the fly were subjected to a correction of 0.5. *P*-values were calculated using the Chi-square test.

In addition, we analyzed the conservation of known phosphorylations observed in cytoskeleton, signaling, and splicing-related proteins, as well as all human phosphoproteins. We observed that the conservation of phosphosites related to insulin signaling proteins and cytoskeletal proteins increased greatly between the speciation of the fish and fly compared with the conservation of all phosphorylations (Figure 6A). However, the conservation of spliceosomal protein phosphorylation was the same as the average among all human proteins. Spliceosomal proteins comprise the core component proteins (complexes A, B, and C) of the spliceosome and common proteins that regulate splicing by interacting with the core component proteins [48]. We compared the conservation of phosphosites in each complex (Figure 6B) and found that the core components that catalyze splicing were more highly conserved than all common proteins. By contrast, the common components exhibited a high increase after fish speciation (Figure 6B). The common components included splicing regulatory factors for heterogeneous nuclear RNP (hnRNP) and serine-arginine-rich (SR) proteins. These proteins are related to the regulation of alternative splicing. Alternative splicing is well known to have increased after the emergence of vertebrates [49, 50]. Thus, the components of the network isolated on the basis of this analysis may affect the diversity of alternative splicing. These results suggest that physiologically important signaling proteins such as spliceosomal proteins expanded during multiple stages of evolution.

We investigated whether the motifs with the sigmoid pattern are related to the expansion of signaling networks. The sigmoid motifs appeared since the worm; thus, we defined the core signaling network as a network that comprised proteins with phosphosites conserved from the yeast to humans. In addition, the network of proteins that interacted with the core signaling network was defined as the additional network that expanded during evolution after the yeast (Additional file 11). Using these networks, we extracted a network that comprised proteins with the sigmoid-type motifs (Figure 6C). Most of the proteins with sigmoid-type motifs appeared to be in the additional network. To validate this bias toward the additional network among the sigmoid-type motifs, we compared it with the random networks. As a result, we found that the proteins with the sigmoid-type phosphomotifs were significantly more likely to be located in the additional network (Figure 6D). In the fly, a small number of proteins with sigmoid -type phosphomotifs were present in the network because most of these proteins are functionally unknown zinc finger proteins and the interactions among them are still unknown. However, both of the proteins related to the fly were present in the additional network, and we could not find them in the core signaling network. These results suggest that the sigmoid-type phosphomotifs could be related to the expansion of the core signaling network.

Furthermore, to investigate the relationship between phosphomotifs and their biological functions, we enriched the phosphomotifs based on their GO biological functions. In general, motifs that increased after speciation events distant from humans were more likely to have correlated functions, whereas motifs that increased after the emergence of mammals lacked correlations with any functions (Additional file 10). These results suggest that the conserved patterns of the phosphomotifs are closed in networks with similar functions.

## Discussion

**Figure 7:**
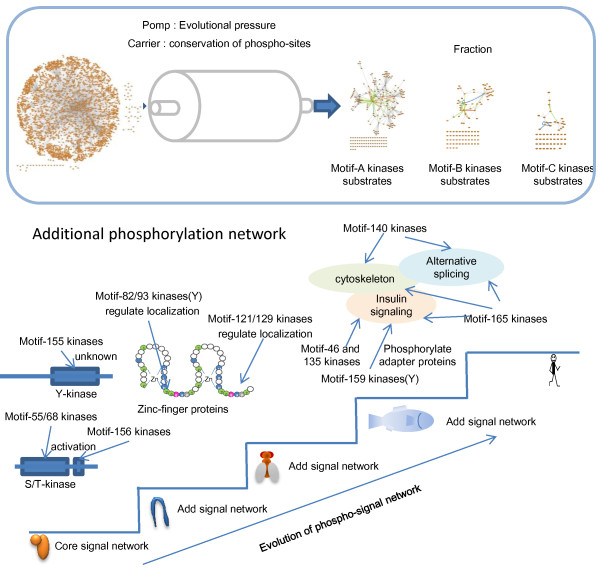
**Evolutionary expansion scenarios for phosphorylation networks.** Schematic representations of our strategy for filtering phosphorylation signaling networks (top). The global signaling networks were fractionated into biologically enriched terms and phosphorylation using subnetworks of kinases and their substrates based on their levels of evolutionary conservation, where the network used a column called “evolution” to represent evolutionary pressure and phosphosite conservations were used as a carrier. Schematic representations of the motifs acquired from a specific species and the functions of the kinases that phosphorylated the motifs (bottom). The biological functions related to the phosphomotifs were acquired through evolution, and they developed in a highly complex mannern.

In this study, we performed comparative analyses of phosphosites using a wide variety of methods and extracted physiologically important phosphorylations on the basis of their evolutionary conservation. Our approach has the advantage that it allowed us to analyze the species with increased phosphomotifs based on their evolutionary conservation patterns, whereas it is difficult for conventional methods to handle these evolutionary patterns because they focus only on phosphosite residues. In addition, our analysis using phosphomotifs allowed us to predict the kinases responsible for regulating the networks. We extracted 178 motifs, including known motifs, which we subjected to a comparative evolutionary analysis. These motifs covered approximately 60% of the serine, threonine, and tyrosine residues observed in the human genome, regardless of the presence/absence of phosphorylation. The remaining 40% residues may not have been covered because of the systematic bias in the phosphorylation information obtained from publicly available databases. Most of the phosphorylation data stored in databases has been produced by large-scale phosphoproteomic analyses. Large-scale phosphoproteomic analysis is a recently developed technology and it is focused primarily on cell growth-related proteomics. The motifs we used included several motifs phosphorylated by CMGC family members related to kinases that regulate cell growth. The efficiency of connecting proteins with the same phosphomotif was 2.5 times higher than that of randomized protein sets, which suggests that our selections of motifs had biological significance. However, the sigmoid-type motifs isolated in this study lacked high connectivity. Thus, the connectivity of sigmoid-type phosphorylations may have been affected less by evolutionary selection, and it may have been determined by other factors. Our approach is similar to the isolation of signaling pathways using chromatography based on the conservation of phosphomotifs under the effects of evolutionary pressure (Figure 7A). We isolated motifs that exhibited dramatic changes between species in separate networks and detected the conservation rates based on nine species, which ranged from yeasts to humans. This analysis showed that the phosphorylation networks acquired by humans since distant speciation events include additional network modules and core signaling networks. This suggests that the added networks are involved with specific functions such as kinase activity regulation (since the speciation of the worm) and control of the localization of zinc finger proteins (since the speciation of the fly), thereby facilitating the spread of information through kinase cascades and transcriptional regulation (Figure 7B). The additional phosphorylation networks were more complex in species closer to humans. The signaling networks added after fish speciation involved alternative splicing-related factors, cytoskeleton regulatory factors, and insulin signaling regulatory factors. Each network was connected via 14-3-3 proteins; therefore, it is assumed that they cooperate with, or are regulated by, closely related kinase family members. Furthermore, specific functional networks could not be obtained for species close to humans, which was obvious in the enrichment analysis (Additional file 10). This was probably because the additional networks were small and they were regulated by diverse signals in species closer to humans. We also observed evolutionary changes relative to humans. It may be possible to increase the number of networks if we use species that are more distant from mammals and increase the total number of species.

In this study, we validated the conservation of motifs and reconstructed pathways based only on known phosphosites. However, there may be differences in the evolutionary conservation of the motifs from all proteins and those that are already known to be phosphorylated. We used 1,771,245 serine/threonine/tyrosine residues in this study. However, we only used 25,631 residues with known phosphorylations in the comparative analysis, which comprised only 1.5% of all residues. To isolate more reliable networks, we performed an analysis that was limited to the known phosphosites, although the number was considerably small. Recently, the volume of phosphosite information has increased dramatically because of the development of phosphoproteomic techniques based on mass spectrometry. These large-scale datasets should allow us to isolate larger numbers of more reliable signal transduction networks.

## Conclusions

In this study, we identified 178 phosphomotifs from publicly available phosphosite information and developed a method for detecting conserved phosphomotifs among species. We found that the highly conserved phosphomotifs in a specific species were related to important cellular processes. Our study suggests that the phosphorylation networks acquired during evolution have added signaling networks to the core signaling networks. Our method can be helpful not only for screening functionally important phosphosites but also for the classification of molecular networks added throughout the evolutionary process.

## Methods

### Definition of phosphomotifs

We downloaded the phosphomotifs defined in PhosphoSitePlus [11] and PhosphoELM [Remark 1] [51] on May 8, 2012. We extracted phosphomotifs validated in high- and low-throughput experiments, and they were manually combined to yield unique motifs. We also extracted known motifs described in previous studies [26] and added them to our phosphomotif dataset. Finally, we obtained 178 known phosphomotifs.

### Conservation of motifs in species ranging from yeasts to humans

We downloaded nine genomes from KEGG [52] (May 8, 2012): *Homo sapiens* (hsa), *Pan troglodytes* (ptr), *Mus musculus* (mmu), *Canis familiaris* (cfa), *Danio rerio* (dre), *Drosophila melanogaster* (dme), *Caenorhabditis elegans* (cel), *Schizosaccharomyces pombe* (spo), and *Saccharomyces cerevisiae* (sce). The three-letter code for each genome is the species identifier defined by KEGG. Orthologous genes among these genomes were defined by KEGG OC [29]. For each ortholog cluster, multiple sequence alignments were constructed by MAFFT [53], which is a freely available, rapid, and reliable tool compared with other alignment tools. MAFFT was run with the “--auto” option, which automatically selected the optimal options. We explored the sequence regions that matched exactly with the known phosphomotifs from all species. We investigated the species conservation with respect to these known and potential phosphorylated sites. In this study, potential phosphosites were defined as all serine/threonine/tyrosine residues in human proteins derived from known phosphosites stored in the databases.

We assumed that the order of evolution for these species corresponded to the general and universal tree, depending on the evolutionary distances from the viewpoint of humans [18, 19]. Therefore, the most basal organisms were the two yeasts, followed by the remaining organisms in the following order: nematodes, fly, fish, and mammals. If an amino acid residue in a protein at the same position as a known phosphorylated site in the orthologous protein in humans did not correspond to the amino acid residues S, T, and Y, we considered that the site was not conserved in the species. However, if the amino acid residue was conserved in a species that was evolutionarily distant from humans, the site was regarded as conserved, even if it was not conserved in the intermediate species. We created sequence logos of 11 residues using the WebLogo application [32], which included known and potential phosphorylated sites in the central positions of these conserved phosphorylation motifs. We also created sequence logos of the motif regions observed in each genome.

### Conservation rate

To compare the conservation of phosphosites, we calculated the conservation rates for the motifs in each species. The conservation rate was defined as the number of motifs conserved in a species divided by the number of motifs observed in the human genome. To confirm that the evolutionary history dramatically affected the conservation rate of sequence motifs, we extracted known motifs where the conservation rate changed >50% between two evolutionarily adjacent species. The conservation rate patterns across all the species were clustered using R (http://www.r-project.org/) based on the Euclidean distance and Ward’s method.

We calculated the conservation rates for all species from yeasts to humans for all known and potential phosphosites. To determine the difference between known and potential phosphosites, we calculated a conservation index (S), which was the sum of the difference between the conservation rate of a phosphosite in a motif (C) and the reference conservation rate (R) of the corresponding amino acid residue obtained from all human proteins. The conservation index was calculated using the following equation:


where G denotes the set of genomes used in the present study, q is each genome selected from G, and Cq and Rq are the conservation rate and the reference conservation rate in q, respectively.

### Enrichment analysis

We extracted GO [44] annotation for the proteins with known phosphorylated sites in the human genome. The annotations at the known motif level were assigned on the basis of the GO biological processes for proteins with the motif. To clarify the functions of the motif, we performed an enrichment analysis using GoMiner [54]. Significant GO annotations were extracted with cutoffs of FDR = 0.01 and *P* < 0.01.

### Interaction networks

To explore the associations between proteins related to a motif, we included their interaction information. We downloaded information related to intermolecular interactions from BioGRID (2.0.58) [30] and STRING (v8.2) [31]. We extracted the interactions of proteins in a known motif from this interaction information. We also compared the interaction networks generated for the motifs with randomly generated networks. We selected the same number of human proteins as those with a motif and extracted the interaction networks of these proteins. This randomization procedure was repeated 100 times. The fold change in a motif was calculated as the number of interactions in a motif divided by the average number of interactions in a randomly generated network. Network visualizations for our data were created using Cytoscape [55].

### Zinc finger analysis

The HMMER programs with the default parameters were used to extract Pfam motifs that corresponded to zinc finger motifs from the genomes [56]. We counted the number of zinc finger motifs in human proteins.

### Functional counts

We counted the conservation levels of the known phosphosites included in our motifs. We also manually extracted human proteins related to the spliceosome, insulin signaling, and the cytoskeleton based on the KO definitions. We counted the conservation levels of these proteins in addition to the level of conservation in all human proteins as a control. We also extracted proteins related to complexes A, B, C, and common components of the spliceosome using the KEGG BRITE functional category and determined their conservation levels.

### Proportion of proteins shared between motifs

We calculated the proportion of proteins shared between two known motifs. We extracted all human proteins with the known motif. The proportion was defined as the number of common proteins in two known motifs divided by the total number of proteins in the two motifs.

### Network expansion of sigmoid-type phosphomotifs

We isolated 585 proteins that possessed phosphosites conserved from yeast (spe and sce) to humans. We defined the interaction network of these proteins as the core signaling network. In addition, we extracted the interactions with the proteins in the core signaling network from BioGRID and STRING, and obtained the additional network for 996 proteins. We extracted the interaction network for the proteins with sigmoid-type phosphomotifs (motifs 55, 56, and 58 for worm; motifs 82, 93, and 121 for fly; and motifs 46, 135, 140, 159 and 165 for fish). We also constructed random interaction networks using the same number of proteins with sigmoid-type phosphomotifs in each genome. This randomization procedure was repeated 100 times. We compared the randomization results and the real counts of proteins with sigmoid-type phosphomotifs in the core signaling network and the additional network.

### Plasmids

cDNAs of 2×C2H2WT, 2×C2H2SN, and 2×C2H2SN were subcloned into pCXN2-mCFP and/or pCXN2-mVenus. pCXN2-mCFP and/or pCXN2-mVenus are expression vectors, which encode monomeric CFP and monomeric Venus, a YFP variant, respectively [57]. The cDNAs of 2×C2H2WT, 2×C2H2SN, and 2×C2H2SN were synthesized by Operon Biotechnology Inc. The pCXN2 vector, which carries a neomycin resistance gene, is derived from pCAGGS.

### Cell culture

The Cos7 cells used in this study were Cos7/E3, a subclone of Cos7 cells established by Y. Fukui. Cos7 cells were maintained in Dulbecco’s modified Eagle’s medium (Sigma, St Louis, MO, USA) supplemented with 10% fetal calf serum. In the transient expression studies, the cells were transfected using Polyfect (Qiagen). The cells were analyzed at 24 h after transfection.

### Imaging of the C2H2 zinc finger motif in living cells

Live cell imaging was performed essentially as previously described. In brief, cells plated on a collagen-coated 35-mm-diameter glass base dish (Asahi Techno Glass Co., Tokyo, Japan) were transfected with C2H2 zinc finger motif expression vectors and imaged every 2 min using an Olympus IX81 inverted microscope (Olympus Optical Co., Tokyo, Japan), which was equipped with a cooled CCD camera, (CoolSNAP HQ; Roper Scientific, Trenton, NJ) and controlled by MetaMorph software (Universal Imaging, West Chester, PA). For the dual-emission ratio imaging of the m1Venus-2×C2H2 and m1CFP-2×C2H2 mutants, we used an excitation filter, i.e., 440AF21 for CFP and S492/18X for YFP, with a dichroic mirror, i.e., 86006bs, and emission filters, i.e., 480AF30 for CFP and 535AF26 for YFP (Omega Optical Inc., Brattleboro, VT). The cells were illuminated with a 75-W xenon lamp through a 12% ND filter (Olympus Optical) and visualized using a 40× oil immersion objective lens. After background subtraction, the ratio of the intensity of the nuclear region relative to the whole cell region was calculated using MetaMorph, which was used to represent the efficiency of the retention of C2H2 motifs in nuclear regions.

## Electronic supplementary material

Additional file 1: **Strategy used to extract the phosphomotifs.** First, we downloaded the phosphomotifs defined in PhosphoSitePlus and PhosphoELM. Next, we extracted preliminary phosphomotifs by clustering, after which the motifs were combined manually into unique motifs. We also extracted the known motifs described in previous studies and included them in our phosphomotif dataset. We obtained 178 original phosphorylation motifs from 434 clusters using the MCL clustering method. (PDF 256 KB)

Additional file 2: **Phosphomotifs identified in this study.** (PDF 83 KB)

Additional file 3: **Scheme showing the comparative evolutionary analysis of the phosphomotifs** **. (A)** The motifs defined by known phosphosites were determined. Multiple alignments were created using orthologs from yeasts to humans based on the phosphoproteins with the motifs. The bar plots show the level of conservation for each species. The sequence logos were generated for the motifs based on the motif sequences in humans. The conservation rates were calculated for the phosphosites. Motifs with specific evolutionary patterns in their conservation rates were screened. **(B)** Examples of conservation index patterns. The red line indicates the conservation rates of phosphomotifs and the blue line indicates the average conservation rates of serine residues in all human proteins. The conservation index was calculated as the sum of the difference between them. A phosphomotif with high conservation rates in humans and a specific species had a high conservation index (top), whereas a motif with a low conservation index was closer to the average pattern (bottom). (PDF 135 KB)

Additional file 4: **List of all phosphomotifs.** Each motif is represented as a bar plot for the conservation rate and as a sequence logo for the sequences in human proteins with the motif pattern. All of the motifs were extracted based on the known human phosphosites. (PDF 1 MB)

Additional file 5: **Conservation of serine/threonine/tyrosine residues.** **(A)** Ratios of serine (S), threonine (T), and tyrosine (Y) residues in all human proteins and those conserved in the genomes of other species. **(B)** Ratios of known phosphorylated STY residues in human proteins relative to those conserved in the genomes of other species. **(C)** The differences in the ratios, which correspond to (A), between the genomes of two neighboring species are shown on the X-axis. **(D)** The differences in the ratios, which correspond to (B), between the genomes of two neighboring species are shown on the X-axis. (PDF 162 KB)

Additional file 6: **Sequence logos for conserved motifs in each genome.** Sequence logos were created for the sequences with phosphosites in each genome. Six representative motifs are represented in the figure. (PDF 241 KB)

Additional file 7: **Conservation of phosphosites.** The conservation levels of each motif are plotted with respect to the phosphorylation sites, proteins, and orthologous groups. The conservation levels of amino acid residues were counted for the phosphosites. The conservation levels of proteins with phosphorylated residues were counted for the proteins, but proteins with multiple phosphorylation sites were not counted repeatedly. The conservation levels of orthologous proteins were counted for the orthologous groups, but multiple paralogous proteins in an orthologous group were not counted repeatedly. (PDF 140 KB)

Additional file 8: **Phosphomotifs acquired from the worm.** **(A)** The worm-specific motifs were motifs 55, 56, and 68. We plotted their conservation levels and generated their sequence logos. The figures created from known phosphomotifs are shown in the top panel; whereas those produced from known and potential phosphomotifs (all S/T/Y residues in human proteins) are shown in the bottom panel. **(B)** Sequence logos of flanking regions for known phosphosites of PDK1 substrates. (PDF 123 KB)

Additional file 9: **Features of phosphosites in zinc finger motifs.** **(A)** Distribution of the lengths of C2H2 motifs in zinc finger proteins. **(B)** Sequence logos of C2H2 motifs for each amino acid length. **(C)** Sequence logos of C2H2 motifs in each genome. (PDF 166 KB)

Additional file 10: **Clustering of enriched GO annotation profiles in phosphomotifs.** The biological processes in the GO annotations were used. The profiles were clustered based on the Euclidean distance and using Ward’s method. The colored cells indicate that annotations were present, whereas white color indicates that they were absent. The functional categories listed on the left side of the table are based on functions related to GO biological processes. (PDF 131 KB)

Additional file 11: **Core and additional signaling networks.** The core signaling network comprised 585 proteins with phosphosites conserved from yeast (sce and spo) to humans. The additional network that interacted with the core signaling network comprised 996 proteins. Red indicates the core signaling network and blue denotes the additional signaling network. (PDF 191 KB)

## Abbreviations

MCL: Markov cluster algorithm
KEGG: Kyoto encyclopedia of genes and genomes
OC: Ortholog cluster
STRING: Search tool for recurring instances of neighbouring genes
CFP: Cyan fluorescent protein
YFP: Yellow Fluorescent protein.
