# Supplementary material for: Elucidation of the evolutionary expansion of phosphorylation signaling networks using comparative phosphomotif analysis
Source: BMC Genomics. 2014 Jul 1;15(1):546. doi: 10.1186/1471-2164-15-546 (PMC4117960; doi:10.1186/1471-2164-15-546)
Supplement: Supplementary file 1 — Additional file 1: Strategy used to extract the phosphomotifs. First, we downloaded the phosphomotifs defined in PhosphoSitePlus and PhosphoELM. Next, we extracted preliminary phosphomotifs by clustering, after which the motifs were combined manually into unique motifs. We also extracted the known motifs described in previous studies and included them in our phosphomotif dataset. We obtained 178 original phosphorylation motifs from 434 clusters using the MCL clustering method. (PDF 256 KB) [file 12864_2014_6298_MOESM1_ESM.pdf]

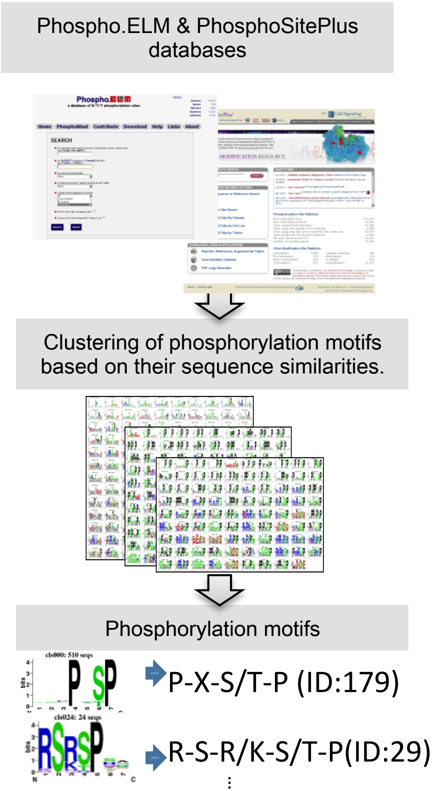
Phospho.ELM & PhosphoSitePlus databases
Clustering of phosphorylation motifs based on their sequence similarities.
Phosphorylation motifs
cls000: 510 seqs
P-X-S/T-P (ID:179)
cls024: 24 seqs
R-S-R/K-S/T-P(ID:29)
⋮
