# Supplementary material for: Elucidation of the evolutionary expansion of phosphorylation signaling networks using comparative phosphomotif analysis
Source: BMC Genomics. 2014 Jul 1;15(1):546. doi: 10.1186/1471-2164-15-546 (PMC4117960; doi:10.1186/1471-2164-15-546)
Supplement: Supplementary file 2 — Additional file 2: Phosphomotifs identified in this study. (PDF 83 KB) [file 12864_2014_6298_MOESM2_ESM.pdf]

Motif ID	Motifs
2	SD/EXD/E
3	S/TPXP
4	PXXS/TP
5	PS/TXP
6	D/ES/TEXE/D
7	PXS/TPP
8	PXS/TPXP
9	PPXS/TP
10	PS/TP
11	PXXS/TPXP
12	S/TP
13	SSP
14	PS/TP/AXP
15	S/TPPP
16	R/Q/KXPS/TP
17	PXPS/TP
18	PS/TPP
19	PXXS/TPP
20	PV/LXS/TP
21	A/S/T/Q/EXS/A/T/Q/ESPA/S/T/V/NA/S/T/V/G
22	PPXS/TPXP
23	D/E/NS/TD/E/NS/TD/N
25	S/TPPG
26	R/K/H/N/QXR/KSXS/TP
27	PS/TPPP
28	PGXSP
29	R/KS/A/G/T/Q/NR/KSP
30	R/KSR/KSP
31	S/TPS
32	PXLSP
33	R/KXXSPXP
34	TPPA/S
35	GXPSP
36	S/A/TXSPR/Q/NS/A/T
38	PXPS/TPP
39	R/K/N/QS/E/D/G/NL/V/I/MSXS/T/A/N/RP
40	PXS/TS/TP
41	GXXSP
42	S/TDS/TES/A/TE
43	R/K/QR/KR/E/H/QS/TPS/E/H/T/VP
44	R/KXPSP
45	R/E/Q/H/KS/G/T/A/EPSPV/S/T/A/NL/I
46	L/V/F/IS/A/N/KS/E/TSPR/Q/EA/S/N
47	S/A/E/N/QS/A/G/Q/TV/F/ISPS/A/Q/TS/A/N/T/V
48	VSP
49	TPPR/K
50	GXGSPXP
51	PXPSS/TXP
52	LXPSP
53	RXRSPS/E/Q/R/TP
54	R/A/KQ/R/KR/KSLS/A/E/G/HP
55	R/K/HT/L/AXTF/C/VCG
56	FGLSR
57	GXXSPR
58	SPXXXP
59	S/TPPXXP
60	PS/TPXXXP
61	S/TPXPP
62	GS/TPXP
63	L/VSPXP
64	S/TPPPP
65	PSPXR/K
66	RSR/KS/TP
67	R/KS/TPXP
68	TF/W/L/V/AC/V/IGT/SP
69	PS/TXPXXP
70	SSPXP
71	SPK/RXK/RK/R
72	RSRSXXP
73	S/TPPXP
74	GS/TPPP
75	GS/TD/ES/AE/DS/AE
76	SPXPXR/K
77	GSPXXXR
78	TPXPR/K
79	RS/TXS/TPS/AP
80	SXPXXP
81	SPEE/D
82	PYXCXXC
83	PSXP
84	TPPRR
85	PSXPXP
86	GSPL
87	SPL
88	SS/V/APGS/T/E/K/QP
89	PSPXXXG
90	GSPXXPP
91	TXPXP
92	TXPXXP
93	PYE/G/VCXXC
94	PSSPPP
95	SS/T/HDS/T/HEE
96	PSPXXPG
97	GTP
98	PXSP
100	D/ED/ED/ESD/E
102	PXXPXSP
103	PXPXPSP
104	PXTP
105	PXPXS/TP
106	PGS/TP
107	PXXSP
108	PXXXXSP
109	PXXXSP
110	PXXPSP
111	PXPSP
112	PPXXSP
113	PPS/TP
114	PGXXSP
115	PXL/I/VSP
116	RR/K/Q/NRR/KL/I/V/WS
117	R/KXRXR/KS/TP
118	PXPPXS/TP
120	R/KR/K/Q/NXXPS/TP
121	HXR/LI/V/LHTG
123	PV/I/LXXSP
125	R/K/Q/NS/G/K/VPXGS/TP
126	RXL/I/V/WSP
127	PXPPS
128	PPPS/TP
129	HXXI/V/L/WHTG
130	PV/I/L/WXXSP
131	LXXSP
132	RS/G/K/VPXGSP
133	RXXSP
134	R/KSP
135	PPPSP
136	RXXPSP
137	PXGXXSP
138	PPPXXS/TP
139	PXPXS/T
140	RE/DRXRS
141	RS/A/KGS/AXSP
142	R/KS/D/E/N/QL/I/FS/G/TS/A/TSP
143	D/ES/TD/ES/TD/E
144	S/A/Q/D/ED/ES/TD/ES/TD/E
145	SXP
146	RXXSXXXL/I/F/V
147	YXXPE/D
148	L/I/V/MY
149	PXYXPXXP
150	YXXPP
152	GXYG
153	D/ER/A/KY
154	GXXXYXXP
155	FXGFSF
156	S/TXK/R
157	D/EXXYXXXXG
158	RXXSXPXL/I/M/V
159	YM/VPM/L/FXP
160	GXXXY
161	L/I/VXXY
162	LXR/KXXS/TXP
163	CG/A/M/S/K/YR/KA/T/S/H/NF/I/L/R/S/CS/YXXXXL/K/N/P/T/W
164	D/EXI/L/M/VYXI/L/M/V
165	RXXSSXS
166	RXS/T
167	RR/KXS/T
168	KRXXS/T
169	RR/S/TXS/TXS/T
170	RXRXXS/T
171	RXXS/TXR
172	V/I/LXR/KXXS/T
173	RXXS/T
174	RXXS/TV
175	S/TXXS/T
176	S/TXXXS
177	S/TXXE
178	S/TPXR/K
179	PXS/TP
180	VXS/TP
181	PES/TP
182	I/E/VYE/GE/D/P/NV/I/L
183	V/I/LYXXP/F
184	D/EXXYXXD/E/A/G/S/T
185	YXXI/L/V/M
186	D/P/S/A/E/NXYV/L/D/E/I/N/P
187	S/TQ
