# Supplementary figures and images for: Elucidation of the evolutionary expansion of phosphorylation signaling networks using comparative phosphomotif analysis
Source: BMC Genomics. 2014 Jul 1;15(1):546. doi: 10.1186/1471-2164-15-546 (PMC4117960; doi:10.1186/1471-2164-15-546)

**A**

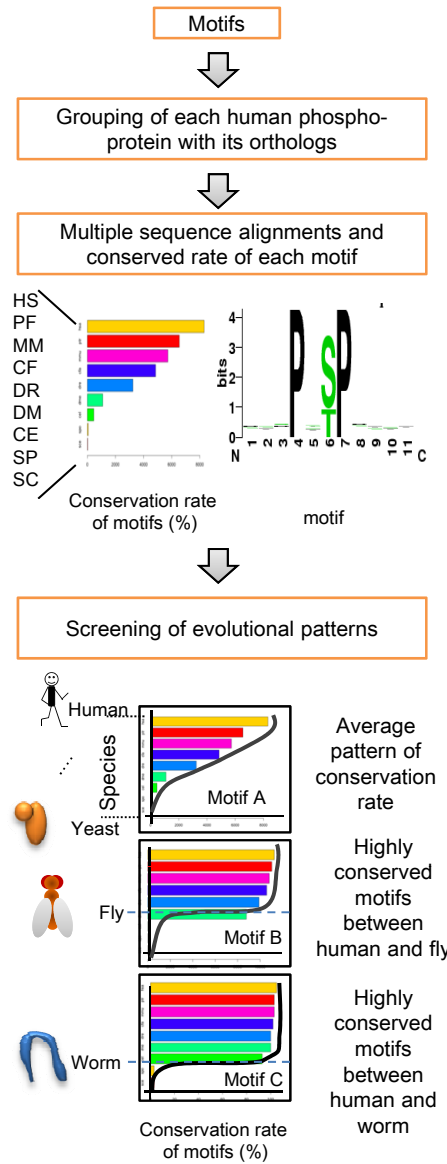

**B**

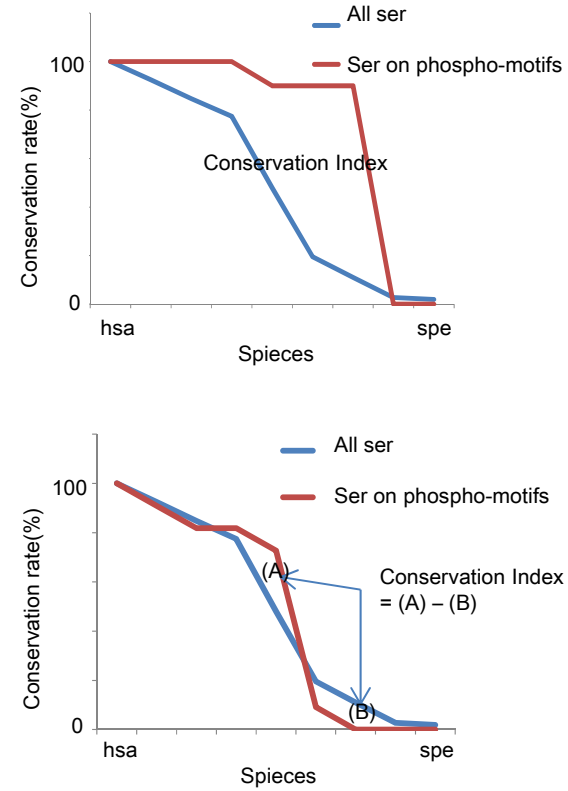

Supplement: Supplementary file 3 — Additional file 3: Scheme showing the comparative evolutionary analysis of the phosphomotifs . (A) The motifs defined by known phosphosites were determined. Multiple alignments were created using orthologs from yeasts to humans based on the phosphoproteins with the motifs. The bar plots show the level of conservation for each species. The sequence logos were generated for the motifs based on the motif sequences in humans. The conservation rates were calculated for the phosphosites. Motifs with specific evolutionary patterns in their conservation rates were screened. (B) Examples of conservation index patterns. The red line indicates the conservation rates of phosphomotifs and the blue line indicates the average conservation rates of serine residues in all human proteins. The conservation index was calculated as the sum of the difference between them. A phosphomotif with high conservation rates in humans and a specific species had a high conservation index (top), whereas a motif with a low conservation index was closer to the average pattern (bottom). (PDF 135 KB) [file 12864_2014_6298_MOESM3_ESM.pdf]

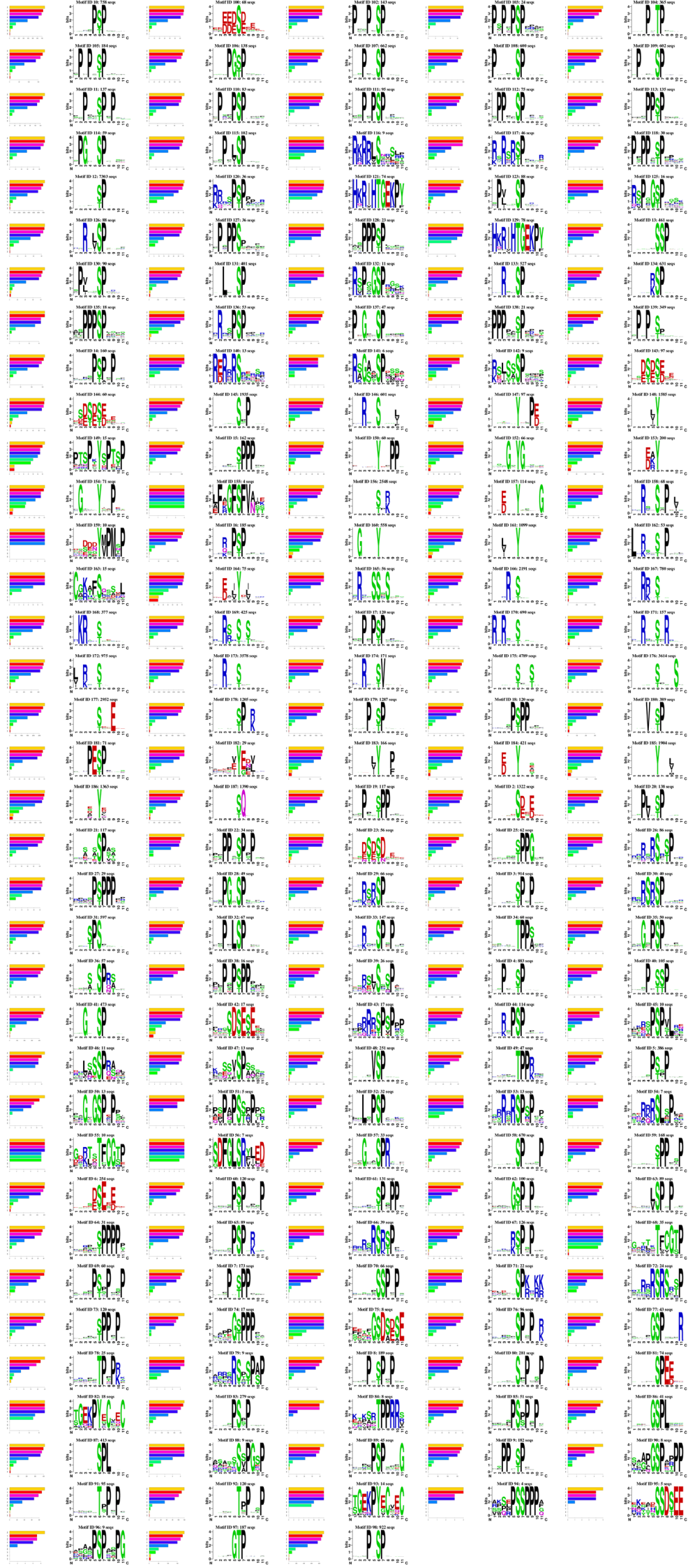

Supplement: Supplementary file 4 — Additional file 4: List of all phosphomotifs. Each motif is represented as a bar plot for the conservation rate and as a sequence logo for the sequences in human proteins with the motif pattern. All of the motifs were extracted based on the known human phosphosites. (PDF 1 MB) [file 12864_2014_6298_MOESM4_ESM.pdf]

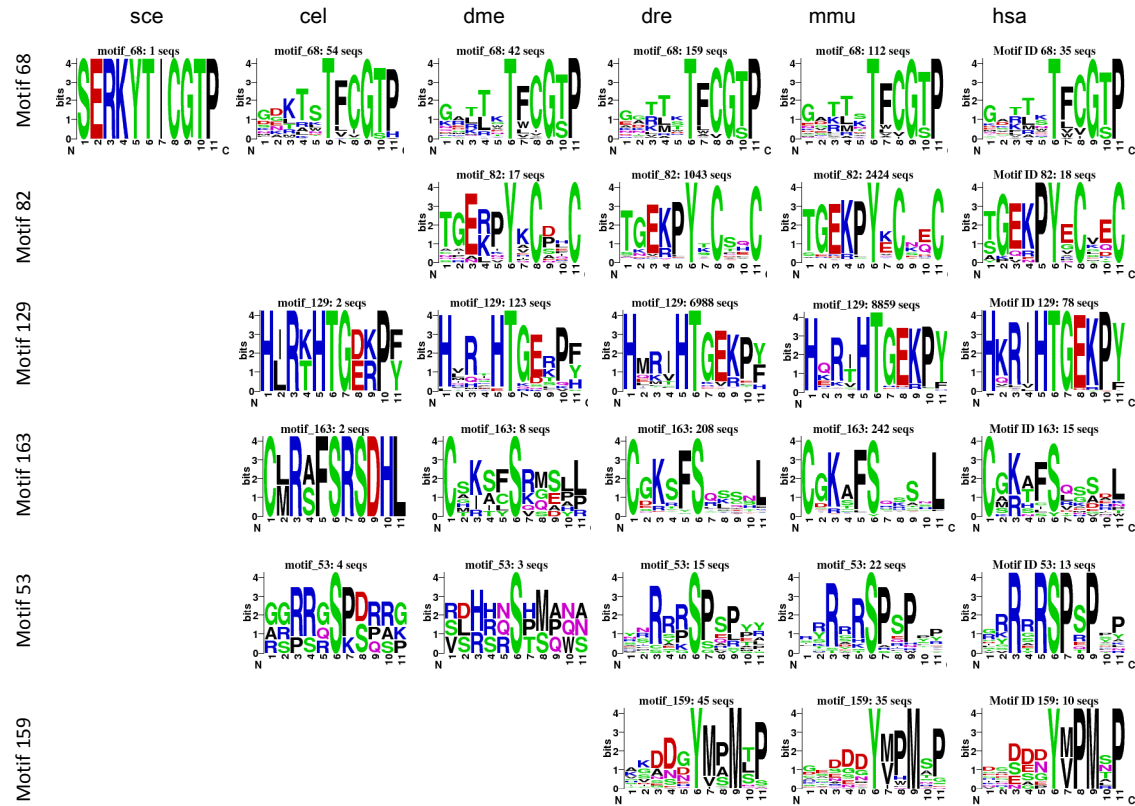

Supplement: Supplementary file 6 — Additional file 6: Sequence logos for conserved motifs in each genome. Sequence logos were created for the sequences with phosphosites in each genome. Six representative motifs are represented in the figure. (PDF 241 KB) [file 12864_2014_6298_MOESM6_ESM.pdf]

Phospho sites

proteins

Orthologue groups

Motif 68

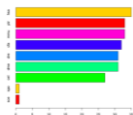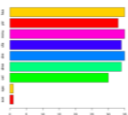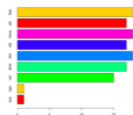

Motif 82

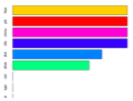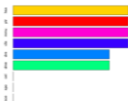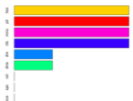

Motif 129

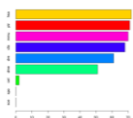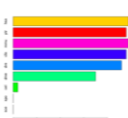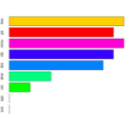

Motif 163

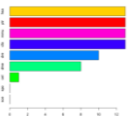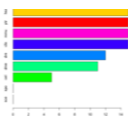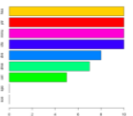

Motif 53

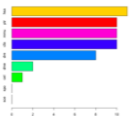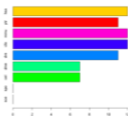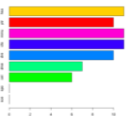

Motif 159

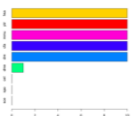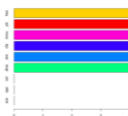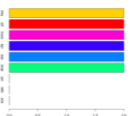

Supplement: Supplementary file 7 — Additional file 7: Conservation of phosphosites. The conservation levels of each motif are plotted with respect to the phosphorylation sites, proteins, and orthologous groups. The conservation levels of amino acid residues were counted for the phosphosites. The conservation levels of proteins with phosphorylated residues were counted for the proteins, but proteins with multiple phosphorylation sites were not counted repeatedly. The conservation levels of orthologous proteins were counted for the orthologous groups, but multiple paralogous proteins in an orthologous group were not counted repeatedly. (PDF 140 KB) [file 12864_2014_6298_MOESM7_ESM.pdf]

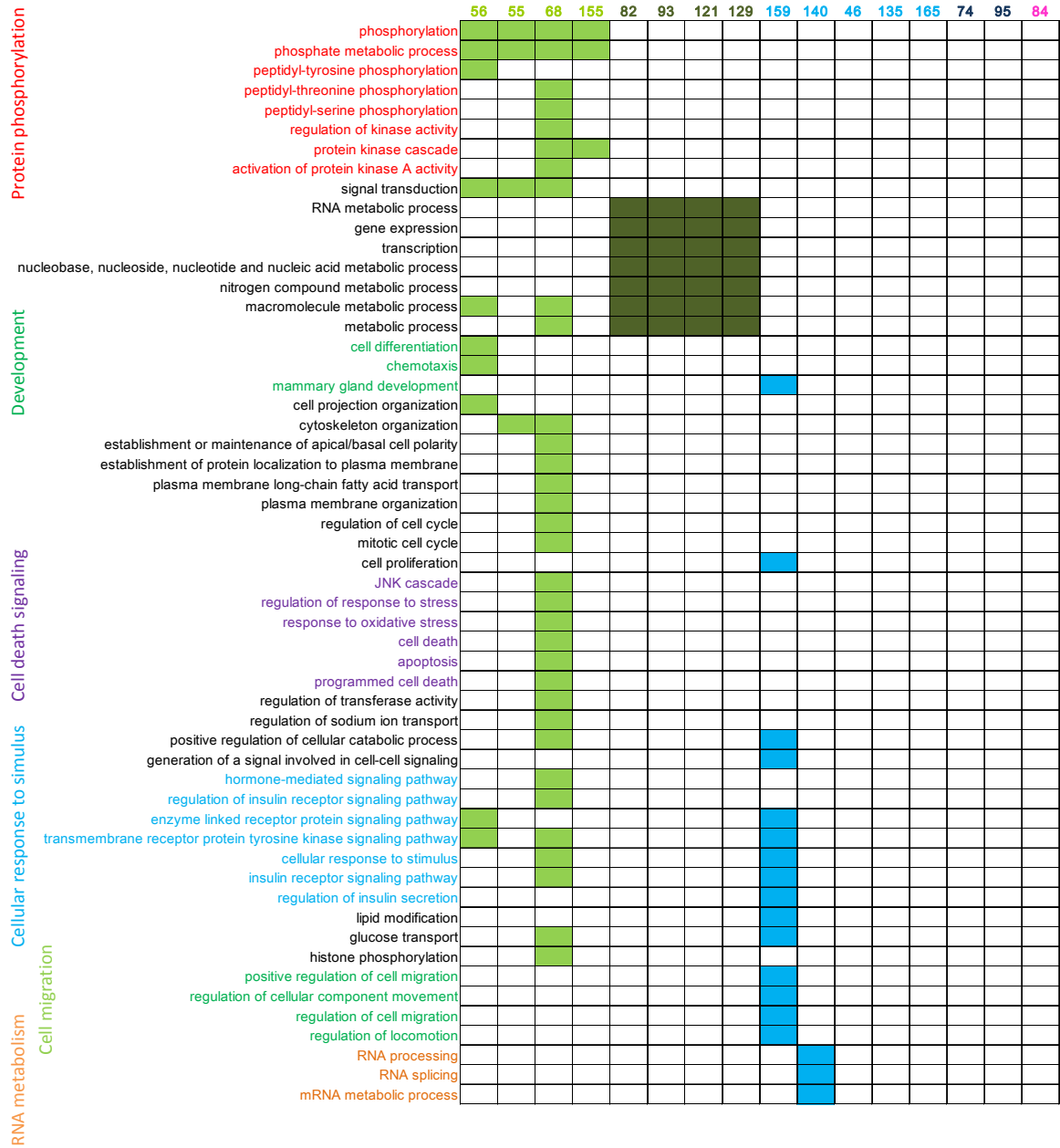

Supplement: Supplementary file 10 — Additional file 10: Clustering of enriched GO annotation profiles in phosphomotifs. The biological processes in the GO annotations were used. The profiles were clustered based on the Euclidean distance and using Ward’s method. The colored cells indicate that annotations were present, whereas white color indicates that they were absent. The functional categories listed on the left side of the table are based on functions related to GO biological processes. (PDF 131 KB) [file 12864_2014_6298_MOESM10_ESM.pdf]

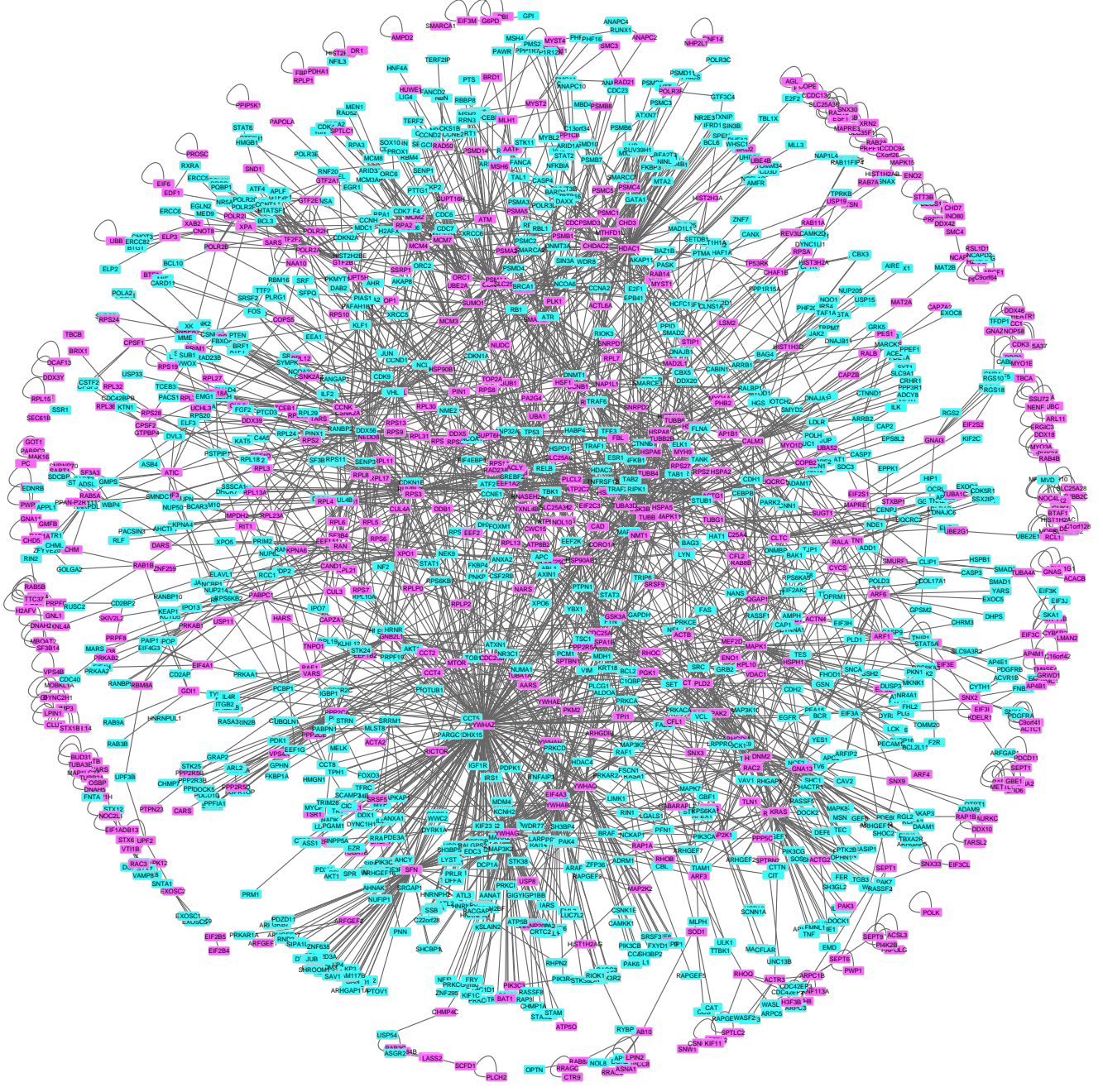

Supplement: Supplementary file 11 — Additional file 11: Core and additional signaling networks. The core signaling network comprised 585 proteins with phosphosites conserved from yeast (sce and spo) to humans. The additional network that interacted with the core signaling network comprised 996 proteins. Red indicates the core signaling network and blue denotes the additional signaling network. (PDF 191 KB) [file 12864_2014_6298_MOESM11_ESM.pdf]
