# Supplementary material for: Elucidation of the evolutionary expansion of phosphorylation signaling networks using comparative phosphomotif analysis
Source: BMC Genomics. 2014 Jul 1;15(1):546. doi: 10.1186/1471-2164-15-546 (PMC4117960; doi:10.1186/1471-2164-15-546)
Supplement: Supplementary file 5 — Additional file 5: Conservation of serine/threonine/tyrosine residues. (A) Ratios of serine (S), threonine (T), and tyrosine (Y) residues in all human proteins and those conserved in the genomes of other species. (B) Ratios of known phosphorylated STY residues in human proteins relative to those conserved in the genomes of other species. (C) The differences in the ratios, which correspond to (A), between the genomes of two neighboring species are shown on the X-axis. (D) The differences in the ratios, which correspond to (B), between the genomes of two neighboring species are shown on the X-axis. (PDF 162 KB) [file 12864_2014_6298_MOESM5_ESM.pdf]

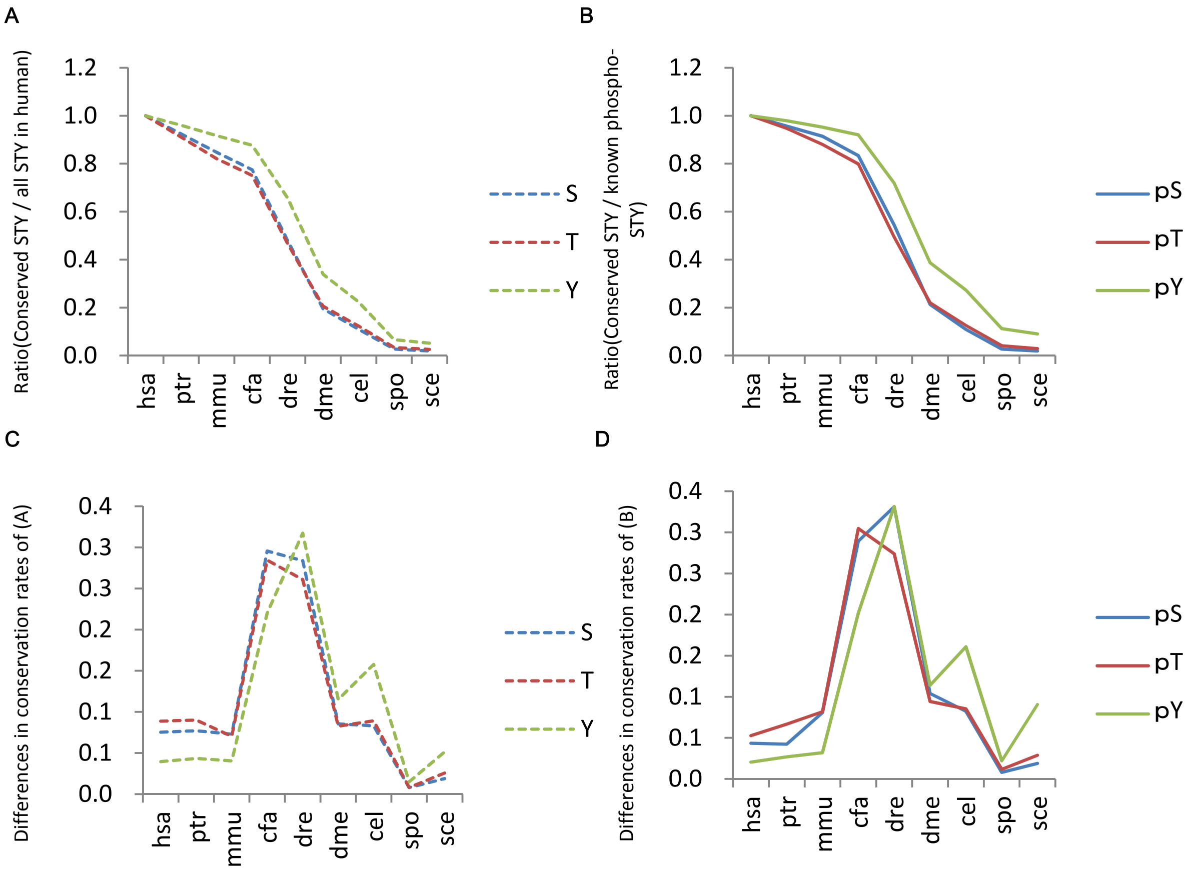

A
Ratio(Conserved STY / all STY in human)
1.2
1.0
0.8
0.6
0.4
0.2
0.0
hsa
ptr
mmu
cfa
dre
dme
cel
spo
sce
S
T
Y
B
Ratio(Conserved STY / known phospho-STY)
1.2
1.0
0.8
0.6
0.4
0.2
0.0
hsa
ptr
mmu
cfa
dre
dme
cel
spo
sce
pS
pT
pY
C
Differences in conservation rates of (A)
0.4
0.3
0.3
0.2
0.2
0.1
0.1
0.0
hsa
ptr
mmu
cfa
dre
dme
cel
spo
sce
S
T
Y
D
Differences in conservation rates of (B)
0.4
0.3
0.3
0.2
0.2
0.1
0.1
0.0
hsa
ptr
mmu
cfa
dre
dme
cel
spo
sce
pS
pT
pY
