# Supplementary material for: Elucidation of the evolutionary expansion of phosphorylation signaling networks using comparative phosphomotif analysis
Source: BMC Genomics. 2014 Jul 1;15(1):546. doi: 10.1186/1471-2164-15-546 (PMC4117960; doi:10.1186/1471-2164-15-546)

A

Known phosphorylation motifs

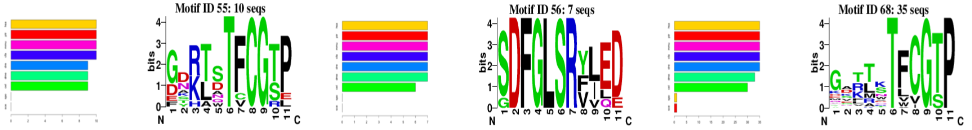

Known and potential phosphorylation motifs

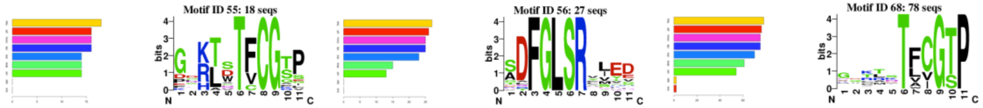

B

Substrate sequence logo of PDK1

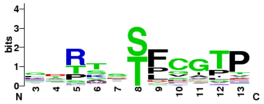

Supplement: Supplementary file 8 — Additional file 8: Phosphomotifs acquired from the worm. (A) The worm-specific motifs were motifs 55, 56, and 68. We plotted their conservation levels and generated their sequence logos. The figures created from known phosphomotifs are shown in the top panel; whereas those produced from known and potential phosphomotifs (all S/T/Y residues in human proteins) are shown in the bottom panel. (B) Sequence logos of flanking regions for known phosphosites of PDK1 substrates. (PDF 123 KB) [file 12864_2014_6298_MOESM8_ESM.pdf]
