# Supplementary material for: Elucidation of the evolutionary expansion of phosphorylation signaling networks using comparative phosphomotif analysis
Source: BMC Genomics. 2014 Jul 1;15(1):546. doi: 10.1186/1471-2164-15-546 (PMC4117960; doi:10.1186/1471-2164-15-546)
Supplement: Supplementary file 9 — Additional file 9: Features of phosphosites in zinc finger motifs. (A) Distribution of the lengths of C2H2 motifs in zinc finger proteins. (B) Sequence logos of C2H2 motifs for each amino acid length. (C) Sequence logos of C2H2 motifs in each genome. (PDF 166 KB) [file 12864_2014_6298_MOESM9_ESM.pdf]

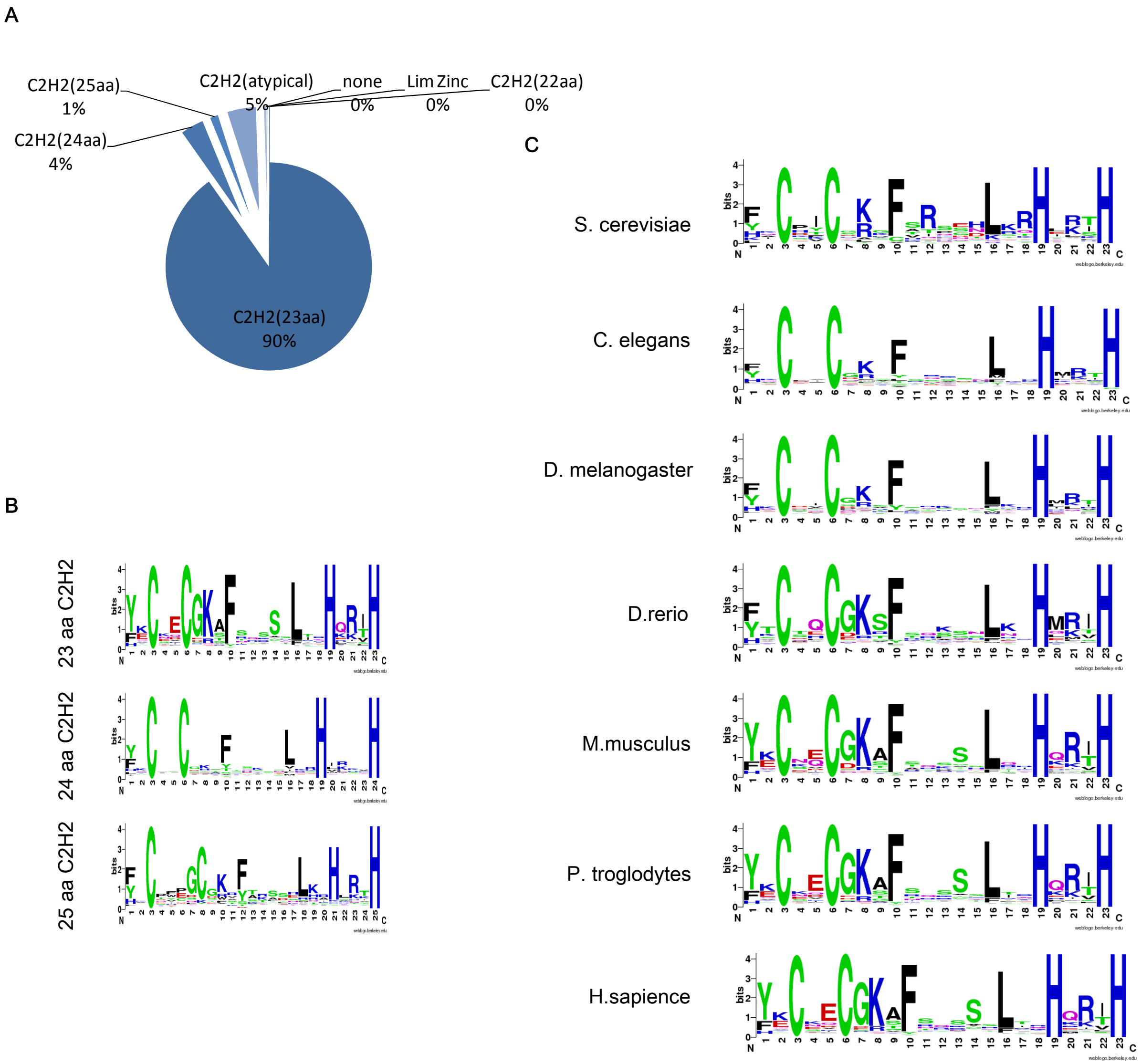
A
C2H2(25aa)
1%
C2H2(atypical)
5%
none
0%
Lim Zinc
0%
C2H2(22aa)
0%
C2H2(24aa)
4%
C2H2(23aa)
90%
B
23 aa C2H2
24 aa C2H2
25 aa C2H2
C
S. cerevisiae
C. elegans
D. melanogaster
D.rerio
M.musculus
P. troglodytes
H.sapience
